# Exploring adolescent participation in football: a gender-differentiated structural equation model based on the theory of planned behavior

**DOI:** 10.3389/fpsyg.2024.1387420

**Published:** 2024-07-25

**Authors:** Xiancheng Zeng, Wei He

**Affiliations:** ^1^Graduate School, Guangzhou Sport University, Guangzhou, China; ^2^Department of Education and Psychology, Guangzhou Sport University, Guangzhou, China

**Keywords:** adolescents, football participation, gender differences, structural equation modeling, theory of planned behavior

## Abstract

This paper explores the intersection of gender and sports participation in adolescence, focusing on traditionally male-dominated sports like football. We aim to develop a structural model based on the Theory of Planned Behavior (TPB) to examine gender-differentiated patterns and factors influencing adolescent participation in football. We analyzes data from 1,147 adolescents using Structural Equation Modeling (SEM). The results indicate that a blend of attitudes, subjective norms, perceived behavioral control, and past behavior effectively predicts adolescents’ willingness to engage in football. Moreover, the study investigates the roles of perceived behavioral control, past behavior, and behavioral intentions in actual football participation, uncovering significant gender disparities in the progression from past behavior to intentions and subsequent behavior. This research highlights the complex role of gender in football participation and provides strategic insights for increasing girls’ involvement in the sport. Our study sets the stage for future research on enhancing girls’ participation in football.

## 1 Introduction

Football, distinguished by its passion, challenge, and global appeal, is universally recognized as one of the most popular sports (Eisenberg, 2006). This sport is not only a spectacle for spectators but also imparts significant health benefits to its participants. Extensive research has underscored the association of football with a myriad of physical and psychological advantages. These benefits range from enhanced physical fitness, cardiovascular health, and motor coordination (Krustrup et al., 2010, 2014; Oja et al., 2015) to the promotion of mental well-being and cognitive function (Battaglia et al., 2013; Eime et al., 2013; McGlynn et al., 2020; Iverson et al., 2021). Moreover, the sport contributes to social skill development due to its inherent nature of team interaction and cooperation (Friedrich and Mason, 2017).

In addition to individual health benefits, football has been recognized for its role in public health initiatives and rehabilitation programs. In the UK, for instance, football has been incorporated into strategies aimed at enhancing public health and has been effectively utilized in various rehabilitation interventions (Pringle et al., 2014; Magee et al., 2015; McKeown et al., 2015; Such et al., 2020).

Parallel to these developments, football’s popularity in China has seen a significant upswing, particularly among the youth. This surge in interest aligns with recent policy reforms and the introduction of innovative training models (Peng et al., 2022). The Chinese government has implemented measures to promote widespread participation in football. These include integrating football into school curricula and supporting youth football through competitions and training programs. In the Medium- and long-term development plan for football in China (2016–2050), by 2030, the aim is to establish 50,000 schools with specialized football programs, highlighting the sport’s unique role in nurturing well-rounded individuals. The role of physical education in fostering a love for sports, including football, is pivotal (Dismore et al., 2010). However, the impact of these enhanced opportunities for football exposure within school curricula on students’ motivation to engage further in the sport remains an area ripe for exploration.

Building on the notion of exploring the impact of enhanced opportunities for football exposure within school curricula on student motivation, this study endeavors to investigate adolescent participation in football, focusing on the gender-differentiated patterns that emerge. Utilizing the Theory of Planned Behavior (TPB) as the foundational framework, the research aims to develop a gender-specific structural model to deeply understand the factors influencing football participation among young individuals. This approach will provide insights into the motivational dynamics of adolescent sports participation, with a particular emphasis on the interplay of gender-specific factors within the context of football.

## 2 Literature review

### 2.1 Gender gap in football participation

The gender gap in sports participation, particularly in gender-stereotyped sports such as football and dance, reflects significant disparities in physical activity engagement between boys and girls. This phenomenon is underpinned by various factors, as evidenced by research from scholars like Chalabaev et al. (2013) and Colley et al. (2005). Boys generally exhibit more positive attitudes towards sports, participate more frequently, and maintain higher levels of physical activity compared to girls (Redelius et al., 2009).

A critical aspect of addressing this gender gap involves understanding the underlying causes of lower participation rates among girls in sports like football. Research in this area suggests that both external and internal factors play a role. External factors encompass social, familial, and peer influences. Socially, there is a prevalent preference for men’s football, which is often seen as more competitive and entertaining. This bias is reflected in the greater volume of research focusing on men’s football (Darongkamas et al., 2011; Robertson et al., 2013). Additionally, cultural, religious, and economic barriers in certain regions can impede girls’ participation in sports (Elliott et al., 2019). From a familial perspective, a tendency exists to value boys’ sports participation over girls’, negatively impacting girls’ motivation and involvement in sports (Fredricks and Eccles, 2005). Peer dynamics also play a critical role, studies show that girls face increased pressure and negative feedback when playing sports like football with boys, which can diminish their enthusiasm and persistence (Soler, 2014).

Internally, factors contributing to girls’ low participation in sports such as football have been examined through various theoretical lenses, including Stereotype Threat Theory, Identity Theory, and Constructivism Theory. Gender stereotypes significantly influence girls’ participation, affecting their self-evaluation, identity formation, cognitive abilities, and perceived proficiency in sports. For instance, concerns about identity and self-image can lead girls to shy away from sports perceived as aggressive (Jeanes, 2011). Gender stereotypes can also lead to more negative self-evaluations and perceptions of physical ability in girls, increasing psychological pressure and potentially impacting performance (Bonnot and Croizet, 2007; Flore and Wicherts, 2015). Studies have demonstrated that girls who internalize football-related stereotypes perform less well in skills like dribbling or shooting after psychological interventions (Chalabaev et al., 2009). This reduced performance can make it challenging for them to experience a sense of fluency and enjoyment in football activities (Hermann and Vollmeyer, 2016), which in turn affects their motivation and long-term participation in the sport (Elliott et al., 2019).

In summary, the gender gap in sports participation, especially in stereotyped sports like football, is a multifaceted issue influenced by external social, familial, and peer factors, as well as internal psychological mechanisms rooted in gender stereotypes and self-perception. Addressing these barriers is crucial for increasing girls’ participation and enjoyment in sports.

### 2.2 Theory of planned behavior in adolescent sports participation

The Theory of Planned Behavior (TPB), as posited by Ajzen (1991, 2011, 2020), stands as a prominent social cognitive model in the realms of health and sports psychology. Renowned for its widespread application, TPB is frequently cited and recognized as one of the most influential models for predicting human social behavior. It provides an extensive framework for examining the determinants of adolescent sports participation, offering insights into how behavioral intentions are shaped by attitudes, subjective norms, and perceived behavioral control.

Recent research has extensively explored the Theory of Planned Behavior (TPB) in the context of sports participation, particularly focusing on adolescent engagement and gender differences. This body of work has progressively built a comprehensive understanding of the various factors influencing youth involvement in sports.

Kanters et al. (2008) initiated this exploration by examining the impact of alternative sports delivery models, such as school intramural sports, on adolescents. Utilizing TPB, they identified key factors influencing youth sports participation and observed significant variances in attitude, subjective norms, and perceived behavioral control across different sporting groups. Building on this foundation, Beville et al. (2014) applied TPB to analyze gender disparities in leisure time physical activity (LTPA) among college students. Their study revealed gender-specific influencers of LTPA, with attitude, intention, self-efficacy, body mass index, and sports participation being significant for females, and intention emerging as the dominant factor for males. This highlighted the need for gender-specific strategies to boost LTPA participation.

To further refine the TPB model, Gourlan et al. (2019) incorporated additional elements such as planning and perceived built environment. Their research underscored the crucial role of subjective norms and attitudes in enhancing the link between perceived behavioral control and intentions. Hopkins et al. (2022) narrowed the focus to female adolescents, exploring personal factors like self-perceptions and desirable outcomes in sports participation. They advocated for the use of TPB in future research to better comprehend and predict sports participation drivers in this demographic.

Zhang et al. (2024) broadened the application of TPB, investigating its constructs’ influence on leisure sports participation among Chinese adolescents. Their findings indicated that attitude and perceived behavioral control positively affect participation intentions, while subjective norm is associated with non-self-determined regulation forms. Additionally, their study illuminated the intricate relationship between the physical environment, TPB constructs, habit strength, and adherence to movement guidelines.

Expanding the domain of TPB, Wang and Zhang (2015) discovered that self-efficacy significantly impacts both intention and behavior. Complementing this perspective, Zhang et al. (2020) concentrated on psychological interventions among Chinese adolescents. The study noted marked improvements in perceived behavioral control, exercise intention, and self-efficacy, which translated into enhanced physical activity duration and intensity in the intervention group, thereby highlighting the efficacy of interventions aimed at boosting adolescent self-efficacy in promoting physical activity.

Collectively, these studies demonstrate the multifaceted role of TPB in understanding and influencing sports participation, offering critical insights into how various psychological and environmental factors impact youth engagement in physical activities across different demographics and cultural contexts.

### 2.3 Augmenting TPB with additional predictors

The Theory of Planned Behavior (TPB) is recognized as a robust model for predicting individual intentions and actions. However, its capability to account for certain behavioral variations is limited. To enhance the explanatory power of TPB, scholars such as Conner and Armitage (1998) have suggested the integration of additional predictive variables into the framework. Ajzen (1991, 2020), the proponent of TPB, advocates for such extensions, provided they are theoretically justified. Among the proposed enhancements are the inclusion of moral norms (Rivis et al., 2009), anticipated regret (Conner et al., 2006; Sandberg et al., 2016), and constructs from health psychology (Blue, 2007; Rise et al., 2010), which are particularly pertinent in the context of health-related behaviors.

Furthermore, the role of past behavior as a significant predictor has gained acknowledgment (Ajzen and Fishbein, 2005). Research conducted by Hagger et al. (2002) demonstrated that past behavior substantially contributes to the formation of behavioral intentions and the execution of actual behavior. In line with these findings, a study by Wing Kwan et al. (2009), involving a sample of Canadian university students, confirmed the predictive value of past behavior in determining future actions, including sports participation. These insights underscore the dynamic and evolving nature of the TPB model, highlighting its adaptability and enduring relevance across various domains of behavioral research.

## 3 Research model and hypothesis development

Drawing on the Theory of Planned Behavior (Ajzen, 1991), this study asserts that attitudes, subjective norms, and perceived behavioral control significantly influence both behavioral intentions and actual participation in football. Research indicates that attitudes are a highly predictive factor of intentions, as evidenced by prior studies (Ajzen, 1991; Cheon et al., 2012). While some research suggests that subjective norms are less predictive of intentions compared to attitudes and perceived behavioral control (Hagger et al., 2002; Plotnikoff et al., 2011), they still serve as a crucial proximal predictor of intentions. Furthermore, perceived behavioral control is posited to directly influence intentions, reflecting an individual’s control over the resources and opportunities necessary for performing a behavior (Ajzen and Madden, 1986; Hagger et al., 2002). This relationship has been substantiated across various studies (Sheeran et al., 2003). Based on these insights, we hypothesize:

*H1a*: Attitudes towards football positively influence students’ intention to play.

*H1b*: Subjective norms related to football positively influence students’ intention to play.

*H1c*: Perceived behavioral control positively influence students’ intention to play football.

*H1d*: A strong intention to play football leads to increased participation.

Following Ajzen’s (2020) updated model, we posit significant pathways from behavioral beliefs and outcome evaluations to attitudes, and from normative beliefs and compliance motivations to subjective norms, as well as from control beliefs and perceived powers to perceived behavioral control. Research indicates that behavioral beliefs are predictive of attitudes, suggesting that attitudes encapsulate an individual’s assessment of the effectiveness of a target behavior in producing desired outcomes, as well as the value placed on these outcomes (Downs and Hausenblas, 2003; Cheon et al., 2012). Thus, outcome evaluations are likely to influence attitudes significantly (Hagger et al., 2002). In terms of normative beliefs and compliance motivations, studies have demonstrated that normative beliefs, particularly lower-order factors, can predict subjective norms, which are essentially the individual’s perception of societal pressures and their motivation to conform (Armitage and Conner, 2001; Downs and Hausenblas, 2003; Cheon et al., 2012). Additionally, control beliefs have been shown to significantly influence perceived behavioral control, which reflects an individual’s evaluation of their capability and resources to perform a behavior (Armitage and Conner, 2001; Downs and Hausenblas, 2003; Cheon et al., 2012). This suggests a direct correlation between an individual’s perceived ability and their perceived behavioral control (Hagger et al., 2002). Based on these insights, we hypothesize:

*H2a*: Beliefs about the benefits of playing football (behavior beliefs) positively impact attitudes.

*H2b*: Positive outcome evaluations enhance attitudes towards football.

*H2c*: Normative beliefs about football positively affect subjective norms.

*H2d*: Compliance motivations positively influence subjective norms in football.

*H2e*: Perceived self-efficacy in football (control beliefs) boosts behavioral control.

*H2f*: Perceived ability to overcome football-related barriers (perceived powers) enhances behavioral control.

Evidence suggests that past behavior not only significantly influences future behavior (Hagger et al., 2002; Wing Kwan et al., 2009; Hsieh et al., 2016) but also serves as a reliable predictor of subsequent actions (Ajzen and Fishbein, 2005). Regarding the link between intentions and actual behavior, numerous studies have affirmed that intentions are potent predictors of behavior (Norman and Conner, 2005; Milosis et al., 2015). Based on these insights, we hypothesize:

*H3a*: Frequent past participation in football correlates with a strong intention to continue playing.

*H3b*: Past involvement in football predicts continued participation.

**Figure 1:**
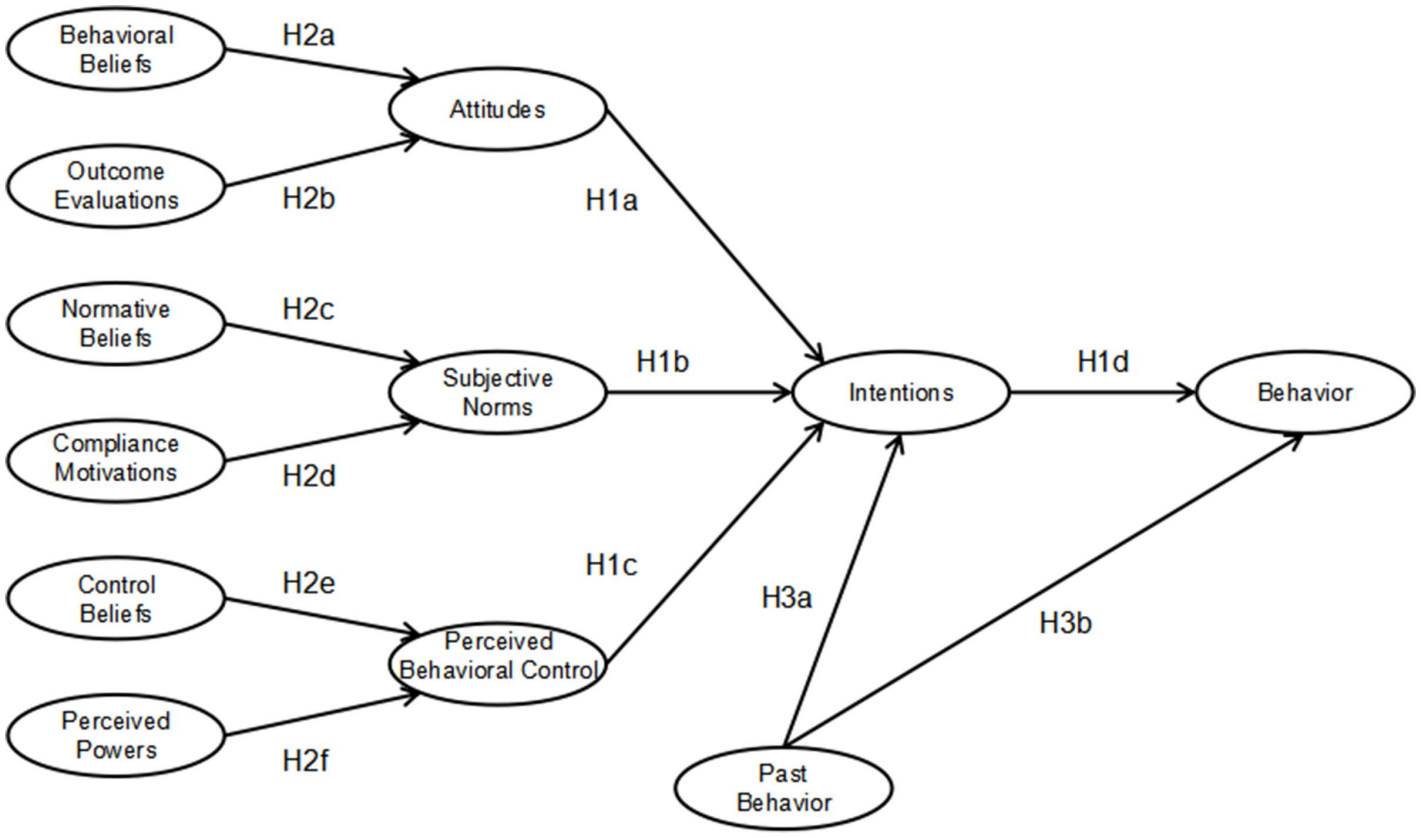
Hypothesis model of theory of planned behavior.

Hypothesis 4 (H4) examines age and gender as moderators in the TPB framework, acknowledging disparities in physical activity engagement (Rhodes et al., 2019) and the moderating roles of age and gender (Hagger et al., 2002). The study proposes that age and gender will moderate the effects of attitudes, subjective norms, perceived behavioral control, and past behavior on the intention to play football and the subsequent participation (see Figure 1).

## 4 Methods

### 4.1 Participants

The study employed a cross-sectional design. It received approval from the university’s ethics committee (No. 2023LCLL-65). Participants in this study came from three primary schools and ten junior high schools in southern China, all of which were involved in a local education organization’s football-specialized school program. Following the acquisition of consent from the parents, the students were instructed to complete a questionnaire. The confidentiality of all student-provided information was assured. A sum of 1,543 questionnaires was amassed. However, to mitigate the risk of acquiescence bias, where respondents might select affirmative responses as a shortcut, potentially not reflecting their true opinions (Krosnick, 1999; Lelkes et al., 2012), portions of the questionnaire exhibiting consistent affirmative answers and any blank questionnaires were omitted from the analysis. The final sample comprised 1,147 participants, with an age range of 8 to 16 years (mean age = 12.52 years, SD = 1.506). Of these, 556 were male.

### 4.2 Development of measurement tools

Ajzen (2020) highlighted the lack of a standardized questionnaire for the Theory of Planned Behavior (TPB). Building on the foundational work by Ajzen (1991, 2002, 2020) and others (Nache et al., 2005; Dou et al., 2022), this study developed a TPB questionnaire specifically tailored for Chinese adolescents’ participation in soccer. To ensure comprehension among younger participants, interviews were conducted with primary school students to verify their understanding of the questionnaire items (Liang et al., 2014). The final questionnaire consists of 33 items, allocating three items to each TPB construct (Iacobucci, 2009). Responses are measured on a 7-point Likert scale, ranging from “totally disagree” to “totally agree,” with prompts such as, “For me, football is very important.” Higher scores reflect more positive attitudes towards football. Additionally, the questionnaire includes items on past behavior (e.g., “In the past 7 days, how many days did you participate in football?”) and demographic details. The scale’s internal consistency is robust (Macdonald’s ω = 0.804–0.914), and both the Average Variance Extracted (AVE = 0.625–0.788) and Construct Reliability (CR = 0.774–0.913) fall within acceptable ranges, indicating the questionnaire’s reliability.

### 4.3 Data analysis

In this study, SPSS 25.0 was employed for descriptive statistics and correlation analysis. Omega values were calculated using JAMOVI 2.3.38 to assess the internal consistency of the subscales. The Structural Equation Model (SEM), based on the Theory of Planned Behavior (Adler et al., 2000), was tested using Mplus 8.3, employing Maximum Likelihood Method (MLM).

## 5 Results

### 5.1 Preliminary analysis

The results of the descriptive statistics and correlation analysis of the research variables are shown in Table 1. The correlation analysis indicates that attitude is significantly correlated with behavioral belief (*r* = 0.841, *p* < 0.001), outcome evaluation (*r* = 0.808, *p* < 0.001). Subjective norm are significantly correlated with normative belief (*r* = 0.818, *p* < 0.001), compliance motivation (*r* = 0.769, *p* < 0.001). Perceived behavior control is significantly correlated with control belief (*r* = 0.868, *p* < 0.001), perceived power (*r* = 0.852, *p* < 0.001). Intention is correlated with attitude (*r* = 0.718, *p* < 0.001), subjective norm (*r* = 0.707, *p* < 0.001), perceived behavior control (*r* = 0.772, *p* < 0.001) and past behavior (*r* = −0.202, *p* < 0.001). Behavior is significantly correlated with behavioral past behavior (*r* = −0.234, *p* < 0.001) and intention (*r* = 0.880, *p* < 0.001).

**Table 1:** Descriptive statistics and correlations of key variables.

Variables	M	SD	Macdonald’ω	AVE	CR	1	2	3	4	5	6	7	8	9	10	11	12	13
Attitudes	4.208	1.826	0.902	0.733	0.846													
Behavioral beliefs	3.747	1.774	0.897	0.733	0.846	0.841^***^												
Outcome evaluations	3.933	1.698	0.852	0.652	0.849	0.808^***^	0.854***											
Subjective norms	3.971	1.780	0.804	0.632	0.774	0.703^***^	0.712^***^	0.744^***^										
Normative beliefs	3.980	1.721	0.830	0.625	0.833	0.683^***^	0.712***	0.754***	0.818^***^									
Compliance motivations	3.981	1.757	0.868	0.668	0.858	0.748***	0.774***	0.785***	0.769^***^	0.810***								
Perceived behavioral control	3.849	1.809	0.900	0.744	0.897	0.694^***^	0.682^***^	0.701^***^	0.712^***^	0.672^***^	0.738^***^							
Control beliefs	4.169	1.754	0.877	0.702	0.876	0.675^***^	0.675***	0.699***	0.710^***^	0.694***	0.733***	0.868^***^						
Perceived intensities	3.782	1.801	0.914	0.788	0.913	0.705^***^	0.703***	0.716***	0.710^***^	0.675***	0.729***	0.852^***^	0.838***					
Past behavior	0.078	0.117	/	/	/	−0.233^***^	−0.175^***^	−0.207^***^	−0.224^***^	−0.201^***^	−0.227^***^	−0.225^***^	−0.207^***^	−0.238^***^				
Intentions	3.648	1.806	0.904	0.763	0.906	0.718^***^	0.698^***^	0.712^***^	0.707^***^	0.680^***^	0.743***	0.772^***^	0.767^***^	0.805***	−0.202^***^			
Behavior	3.430	1.831	0.897	0.740	0.895	0.684^***^	0.669^***^	0.677^***^	0.669***	0.644^***^	0.709^***^	0.734^***^	0.703^***^	0.772^***^	−0.234^***^	0.880^***^		
Age	12.520	1.506	/	/	/	0.104^***^	0.028	0.053	0.097^**^	0.079^**^	0.050	0.041	0.057	0.029	−0.045	0.091^***^	0.104^***^	
Gender	/	/	/	/	/	−0.196^***^	−0.183^***^	−0.175^***^	−0.140^***^	−0.111^***^	−0.168^***^	−0.212^***^	−0.166^***^	−0.182^***^	0.016	−0.170^***^	−0.174^***^	−0.183^***^

^*^p < 0.05, ^**^p < 0.01, ^***^p < 0.001.AVE, average variance extracted, CR, composite reliability.

### 5.2 Gender and age differences in the theory of planned behavior factors

We conducted a MANOVA using SPSS to explore differences in the factors of the Theory of Planned Behavior across gender and age groups (Primary school vs. Secondary school). The results, detailed in Table 2, reveal that by gender, all constructs exhibit differences between boys and girls except for past behavior. By age, there are observable differences between the primary and secondary school groups in attitudes, past behavior, and behavioral intentions, while no differences were found in other constructs.

**Table 2:** Differences in the factors of the theory of planned behavior across gender and age groups.

Variables	Male group	Female group	*F*	*p*	Primary school group	Secondary school group	*F*	*p*
Attitudes	4.576(1.904)	3.862(1.678)	49.251	< 0.001	4.078(1.867)	4.305(1.790)	6.005	0.014
Behavioral beliefs	4.082(1.869)	3.432(1.619)	39.741	< 0.001	3.718(1.801)	3.768(1.755)	0.642	0.423
Outcome evaluations	4.238(1.771)	3.645(1.574)	37.299	< 0.001	3.881(1.676)	3.971(1.714)	1.447	0.229
Subjective norms	4.228(1.860)	3.729(1.667)	26,225	< 0.001	3.870(1.853)	4.046(1.722)	3.598	0.058
Normative beliefs	4.176(1.813)	3.795(1.609)	15.069	< 0.001	3.896(1.763)	4.042(1.687)	2.624	0.106
Compliance motivations	4.213(1.874)	3.622(1.588)	35.514	< 0.001	3.893(1.775)	3.920(1.744)	0.280	0.597
PBC	4.245(1.883)	3.476(1.653)	54.655	< 0.001	3.858(1.867)	3.842(1.765)	0.044	0.834
Control beliefs	4.469(1.794)	3.888(1.668)	35.104	< 0.001	4.158(1.797)	4.178(1.722)	0.209	0.674
Perceived powers	4.119(1.915)	3.465(1.625)	41.011	< 0.001	3.804(1.872)	3.765(1.747)	0.005	0.943
Past behavior	0.077(0.121)	0.080(0.113)	0.328	0.567	0.070(0.132)	0.084(0.104)	4.053	0.044
Intentions	3.966(1.903)	3.350(1.656)	37.890	< 0.001	3.546(1.840)	3.723(1.778)	3.768	0.052
Behavior	3.759(1.956)	3.121(1.831)	38.066	< 0.001	3.293(1.886)	3.531(1.784)	6.323	0.012

PBC, perceived behavioral control.

### 5.3 Hypothesis testing

We conducted structural equation modeling (SEM) to test the proposed hypotheses on our sample. The SEM analysis indicated an adequate fit with the data: χ^2^ = 1602.492, Comparative Fit Index (CFI) = 0.953, Tucker-Lewis Index (TLI) = 0.947, Root Mean Square Error of Approximation (RMSEA) = 0.044, and Standardized Root Mean Square Residual (SRMR) = 0.030. These results suggest that the model adequately represents the observed data.

**Figure 2:**
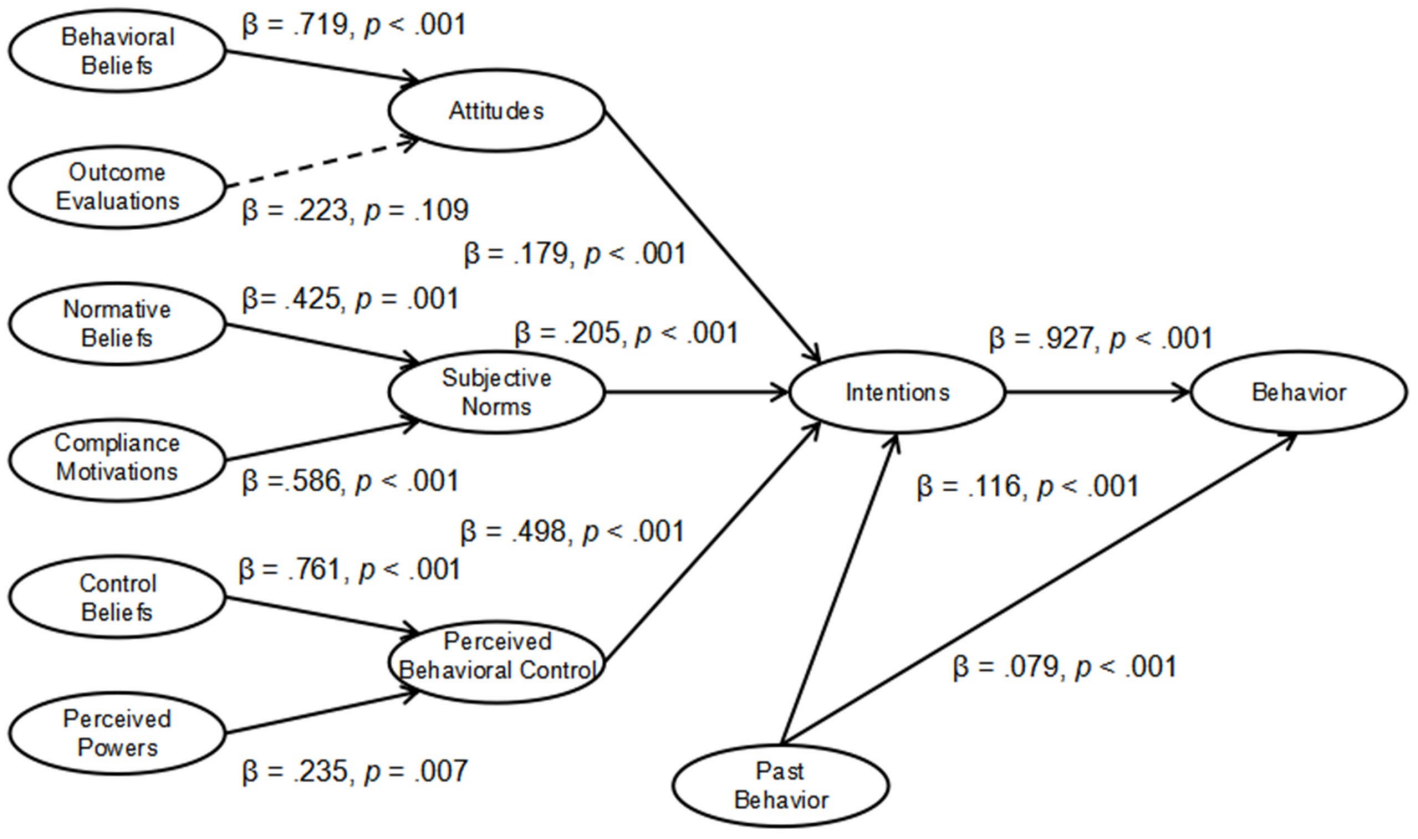
Model of theory of planned behavior. Solid line indicates path is significant. Dashed lines indicate path is not significant.

As illustrated in Table 2 and Figure 2, our findings support the hypothesized relationships. Attitudes (*β* = 0.177, *p* < 0.001), subjective norms (*β* = 0.215, *p* < 0.001), perceived behavioral control (*β* = 0.492, *p* < 0.001), and past behavior (*β* = 0.115, *p* < 0.001) all have a significant impact on intentions, meaning that these factors together contribute to the formation of an individual’s behavioral intentions (for H1a-c, H3a).

Intentions have a very strong predictive effect on behavior (*β* = 0.927, *p* < 0.001), which is consistent with the core assumption of the theory of planned behavior, that is, intentions are the direct precursors to behavior (for H1d). Past behavior also has a significant impact on current behavior (*β* = 0.079, *p* < 0.001), indicating that past behavior can to some extent predict future behavior (for H3b).

Regarding Hypothesis 2 (H2a-f), behavioral beliefs have a very significant effect on attitudes (*β* = 0.693, *p* < 0.001), indicating that behavioral beliefs play an important role in the formation of attitudes. The impact of outcome evaluations on attitudes is not very significant (*β* = 0.249, *p* = 0.075), suggesting that the influence of outcome evaluations on attitudes might be relatively small. Normative beliefs have a significant impact on subjective norms (*β* = 0.465, *p* < 0.001), indicating that normative beliefs are an important factor in forming subjective norms. Compliance motivations also have a significant impact on subjective norms (*β* = 0.543, *p* < 0.001), suggesting that the motivation to comply with others’ expectations is another key factor in forming subjective norms. Control beliefs have a strong influence on perceived behavioral control (*β* = 0.772, *p* < 0.001), showing that control beliefs are very important in an individual’s perception of their ability to control behavior. Perceived power has a significant but weaker effect on perceived behavioral control (*β* = 0.224, *p* = 0.011), indicating that individuals’ perception of their own abilities does impact their perceived behavioral control to some extent.

### 5.4 The moderation role of gender and age group

In the context of the Theory of Planned Behavior (TPB), we recognize gender and age as potential moderating factors that might influence the relationships within the model (Nigg et al., 2009). Since the participants come from elementary and junior high schools, and there is a high level of consistency within each educational stage, we treated age as a dichotomous variable representing the two educational stages. To investigate these moderating effects, we segmented the model to examine the influence of gender and age (educational stage) on seven key pathways: attitudes to behavioral intentions, subjective norms to behavioral intentions, perceived behavioral control to behavioral intentions, past behavior to behavioral intentions, perceived behavioral control to behavior, past behavior to behavior, and behavioral intentions to behavior.

Firstly, we assessed the measurement invariance of TPB constructs with respect to gender and age moderation. As depicted in Table 3, our analysis revealed that under the moderation of gender and age, the configuration, loadings, and scalar invariance of attitudes, perceived behavioral control, and behavioral intentions showed a decrease in Comparative Fit Index (CFI) of less than 0.01. According to Chen (2007), a model fit can be deemed acceptable if the decrease in CFI is below 0.01, the increase in Root Mean Square Error of Approximation (RMSEA) is less than 0.015, and both RMSEA and Standardized Root Mean Square Residual (SRMR) are below 0.08, with CFI above 0.90.

**Table 3:** Measurement invariance of the TPB.

Gender									Age								
Model	Route	χ^2^	df	CFI	△CFI	SRMR	RMAEA	△RMAEA	Model	Route	χ^2^	df	CFI	△CFI	SRMR	RMAEA	△RMAEA
1	M1 Configural invariance	0	0	1	/	0	0	/	4	M1 Configural invariance	0	0	1	/	0	/	/
1	M2 Metric invariance	0.37	2	1	0	0.007	0	0	4	M2 Metric invariance	1.974	2	1	0	0.017	0	0
1	M3 Scalar invariance	8.314	4	0.998	−0.002	0.021	0.043	0.043	4	M3 Scalar invariance	3.975	4	1	0	0.014	0	0
2	M1 Configural invariance	0	0	1	/	0	0	/	5	M1 Configural invariance	0	0	1	/	0	0	/
2	M2 Metric invariance	1.825	2	1	0	0.013	0	0	5	M2 Metric invariance	0.752	2	1	0	0.010	0	0
2	M3 Scalar invariance	4.258	4	1	0	0.013	0	0	5	M3 Scalar invariance	2.29	4	1	0	0.010	0	0
3	M1 Configural invariance	0	0	1	/	0	0	/	6	M1 Configural invariance	0	0	1	/	0	0	/
3	M2 Metric invariance	7.918	2	0.997	−0.003	0.030	0	0.030	6	M2 Metric invariance	1.437	2	1	0	0.013	0	0
3	M3 Scalar invariance	12.927	4	0.995	−0.002	0.027	0	−0.003	6	M3 Scalar invariance	7.345	4	0.999	−0.001	0.016	0.038	0.038

Model 1 is attitudes toward intentions, the moderation of gender.Model 2 is perceived behavioral control toward intentions, the moderation of gender.Model 3 is intentions toward behaviors, the moderation of gender.Model 4 is attitudes toward intentions, the moderation of age.Model 5 is perceived behavioral control toward intentions, the moderation of age.Model 6 is intentions toward behaviors, the moderation of age.

Subsequently, we conducted Structural Equation Modeling (SEM) tests again. With gender as the moderating variable, the SEM analysis indicated an adequate fit: χ^2^ = 32.694, CFI = 0.989, Tucker-Lewis Index (TLI) = 0.966, RMSEA = 0.088, and SRMR = 0.022. When age was the moderating variable, the SEM analysis also showed an adequate fit: χ^2^ = 36.718, CFI = 0.987, TLI = 0.961, RMSEA = 0.094, and SRMR = 0.023. According to Browne and Cudeck (1992), an RMSEA value between 0.08 and 0.10 indicates a moderate fit, suggesting that our model exhibited an adequate fit under both gender and age moderation.

As depicted in Table 4. gender and age moderate the relationship between past behavior to intentions.

**Table 4:** The associations between TPB elements, intention and behavior by gender and age.

	Male/8–12 years old					Female/13–16 years old						Comparison					
Model	Route	B	SE	*p*	95%CI	Model	Route	B	SE	*p*	95%CI	Model	Route	B	SE	*p*	95%CI
1	Attitude → Intentions	0.276	0.047	<0.001	[0.198,0.354]	1	Attitude → Intentions	0.276	0.052	<0.001	[0.191,0.362]	1	Attitude → Intentions	0.003	0.070	0.967	[−0.112,0.118]
1	Subjective norm → Intentions	0.230	0.048	<0.001	[0.152,0.309]	1	Subjective norm → Intentions	0.177	0.042	<0.001	[0.107,0.246]	1	Subjective norm → Intentions	0.060	0.065	0.352	[−0.046,0.166]
1	Perceived behavioral control → Intentions	0.426	0.049	<0.001	[0.346,0.506]	1	Perceived behavioral control → Intentions	0.434	0.054	<0.001	[0.345,0.524]	1	Perceived behavioral control → Intentions	−0.005	0.073	0.948	[−0.125,0.116]
1	Past behavior → Intentions	0.067	0.001	0.006	[0.027,0.107]	1	Past behavior → Intentions	−0.065	0.026	0.011	[−0.107,-0.023]	1	Past behavior → Intentions	2.000	0.531	<0.001	[1.127,2.873]
1	Past behavior → Behavior	−0.066	0.021	0.002	[−0.101,-0.032]	1	Past behavior → Behavior	−0.056	0.022	0.011	[−0.092,-0.020]	1	Past behavior → Behavior	−0.257	0.465	0.581	[−1.021,0.508]
1	Intentions → Behavior	0.869	0.013	<0.001	[0.848,0.889]	1	Intentions → Behavior	0.860	0.015	<0.001	[0.835,0.885]	1	Intentions → Behavior	0.036	0.027	0.179	[−0.008,0.080]
2	Attitude → Intentions	0.303	0.056	<0.001	[0.211,0.395]	2	Attitude → Intentions	0.246	0.045	<0.001	[0.171,0.320]	2	Attitude → Intentions	0.055	0.071	0.442	[−0.063,0.173]
2	Subjective norm → Intentions	0.166	0.045	<0.001	[0.093,0.240]	2	Subjective norm → Intentions	0.236	0.049	<0.001	[0.156,0.316]	2	Subjective norm → Intentions	−0.079	0.067	0.238	[−0.189,0.031]
2	Perceived behavioral control → Intentions	0.445	0.055	<0.001	[0.355,0.535]	2	Perceived behavioral control → Intentions	0.431	0.050	<0.001	[0.349,0.512]	2	Perceived behavioral control → Intentions	0.005	0.073	0.944	[−0.115,0.126]
2	Past behavior → Intentions	0.048	0.028	0.086	[0.002,0.095]	2	Past behavior → Intentions	−0.037	0.022	0.096	[−0.073,0.000]	2	Past behavior → Intentions	1.304	0.546	0.017	[0.406,2.201]
2	Past behavior → Behavior	−0.082	0.024	0.001	[−0.122,-0.043]	2	Past behavior → Behavior	−0.039	0.018	0.029	[−0.069,-0.010]	2	Past behavior → Behavior	−0.504	0.458	0.272	[−1.258,0.250]
2	Intentions → Behavior	0.855	0.015	<0.001	[0.831,0.879]	2	Intentions → Behavior	0.879	0.012	<0.001	[0.859,0.900]	2	Intentions → Behavior	−0.006	0.026	0.807	[−0.049,0.037]

Model 1 is the moderation of gender.Model 2 is the moderation of age.

## 6 Discussion

This research utilized the Theory of Planned Behavior (TPB) to investigate the factors influencing adolescent participation in football, focusing on gender and age as moderating variables. The study revealed significant impacts of behavioral beliefs on attitudes and normative beliefs on subjective norms in shaping football participation among youths. While control beliefs crucially affected perceived behavioral control, their impact was moderate compared to perceived powers. Adolescents’ attitudes, subjective norms, and perceived behavioral control significantly influenced their willingness to participate in football, highlighting the importance of these factors in forming behavioral intentions. Notably, past behaviors also predicted current behaviors, indicating a consistency in individual behavior patterns. Moreover, the study observed that gender and age moderated the relationship between past behavior and behavioral intention, contributing to a deeper understanding of factors affecting youth participation in football and gender differences therein.

### 6.1 The application and expansion of planned behavior theory in football

The study significantly contributes to the understanding of adolescents’ participation in football through the lens of the Theory of Planned Behavior (TPB). This framework, as proposed by Ajzen (1991), has been effectively applied in various contexts (Plotnikoff et al., 2011; Kekäläinen et al., 2022), and our study further expands its applicability in the realm of football. Here, we discuss the implications of our findings in relation to each component of the TPB - attitudes, subjective norms, perceived behavioral control, and behavioral intention, and suggest practical strategies for enhancing youth participation in football.

#### 6.1.1 Attitudes toward football

Our findings reinforce the importance of cultivating positive attitudes towards football among adolescents. Societal, educational, and familial initiatives play a crucial role in introducing the physical and social benefits of football to young people (Oja et al., 2015; Friedrich and Mason, 2017). Such efforts can help in shaping a positive perception of the sport, thereby influencing adolescents’ willingness to participate in football activities.

#### 6.1.2 Influence of subjective norms

Subjective norms, encompassing the support and encouragement from parents, teachers, and peers, are pivotal in shaping adolescents’ football participation. Our results suggest that enhancing these supportive networks can significantly impact youths’ involvement in the sport (Downs and Hausenblas, 2003; Rhodes et al., 2019). This underscores the need for a communal approach in encouraging adolescents to engage in football, emphasizing its benefits for physical and mental well-being.

#### 6.1.3 Perceived behavioral control

The aspect of perceived behavioral control highlights the role of football coaches and physical education teachers in moderating training difficulty, designing engaging activities, and fostering a sense of mastery over football skills (Plotnikoff et al., 2011). Creating an environment where students feel competent and in control is crucial for sustaining their interest and participation in football.

#### 6.1.4 Behavioral intention and actual participation

Lastly, our study illustrates the link between behavioral intention and actual participation in football. Coaches and educators should focus on assisting adolescents in setting realistic football goals and developing actionable plans to achieve them. This guidance is instrumental in translating their intentions into concrete participation in football activities.

In summary, our study expands the application of TPB in understanding and enhancing adolescents’ engagement in football. By addressing each component of the TPB, we can develop comprehensive strategies that promote positive attitudes, strengthen supportive networks, optimize perceived behavioral control, and effectively translate intentions into participation. This holistic approach is vital for fostering a healthier and more active youth through the sport of football.

### 6.2 Equalizing gender perceptions in football

This study critically examined the influence of gender and age as potential moderating factors in adolescents’ engagement in football. This examination is particularly significant considering previous findings that highlight a higher rate of sports participation among boys than girls (Fredricks and Eccles, 2005; Pielichaty, 2015; Wang and Wang, 2015; Pedersen et al., 2019). Although the boys group generally scored higher than the girls group on the variables based on the theory, no differences were found when comparing paths using structural equation modeling. However, our results diverged from these earlier studies, as we did not find significant gender or age disparities in the relationships among attitudes, subjective norms, perceived behavioral control, and behavioral intentions. This could indicate a trend toward equalizing gender perceptions and participation in football.

The lack of observed gender differences in our study, which utilized the Theory of Planned Behavior, might stem from the specific characteristics of the sample, particularly if it is drawn from football-specialized schools. Such schools might have a unique culture or environment that minimizes or masks typical gender differences found in broader populations. Additionally, the effective implementation of the “Campus Football” program in China over 14 years, which included substantial investments in resources such as coach training and infrastructure development, especially emphasizing women’s football, may have influenced our findings. The increase in women’s football matches and modifications to the rules have been shown to increase sports participation (Burton et al., 2011), and the incorporation of simplified rules and game elements in physical education and football training has spiked girls’ interest in the sport (Elliott et al., 2019), leading to an alteration in girls’ perceptions and attitudes towards football.

Furthermore, the comparison between the global achievements of Chinese women’s football and the less ideal performance of men’s football has elevated the social recognition of women’s football in China. The notable successes of women’s football and role models have piqued girls’ interest in the sport, thereby changing the perception of football as not exclusively a male-dominated sport (Martin et al., 2017).

Another factor contributing to our findings could be the self-report nature of the measurements used in the study. Self-report methods, while valuable for gathering subjective data, can sometimes lead to biases or inaccuracies in reporting, potentially obscuring real differences between genders. Cultural norms and societal expectations regarding gender roles in sports can vary widely, and in some contexts, these influences might minimize perceived differences between genders in attitudes and behaviors related to sports.

The recent increase in dedicated physical activity time in schools by the Chinese Ministry of Education (China, 2021), coupled with growing parental concern for their children’s physical health, has led to more support for participation in sports activities. Notably, parental support has been found to significantly promote sports involvement among children, especially girls (Higginson, 1985; Brown et al., 1989; Anderson et al., 2003), potentially reducing gender disparities in sports participant intention.

Finally, peer influence also plays a crucial role in sports participation among adolescents. As adolescents spend considerable time in school environments where peers and teachers significantly impact their development, the enhanced awareness and time dedicated to sports activities, along with an improved overall sports atmosphere, could positively influence girls’ inclination towards sports like football (Prochaska et al., 2002; Bokhorst et al., 2010; Chung et al., 2017; Pluta et al., 2020).

### 6.3 Gender differences in past behavior

The observed differences in the relationship between past behavior and actual behavior underscore a critical gender gap. Notably, even though girls recognize the benefits and acknowledge their own ability to engage in football, their participation rates lag significantly behind those of boys. This discrepancy raises important questions about the underlying factors influencing such trends.

A crucial aspect to consider is the influence of societal and cultural norms on girls’ participation in sports, especially in football. These norms frequently create significant barriers, as girls often face more pronounced social and familial challenges compared to boys. They are typically less encouraged to participate in sports that are traditionally viewed as masculine. This observation is supported by the research of Channon et al. (2016), who discussed how sports reflect and reinforce societal gender norms. In the realm of football, these norms can act as substantial impediments to female participation. Furthermore, Saemi et al. (2023) have highlighted that these influences often operate in implicit ways, making them difficult to capture through explicit self-report measures.

In addition, the impact of gender stereotypes in shaping sports participation is profound. Plaza and Boiché (2017) emphasized the significant role of gender stereotypes and self-perceptions in influencing dropout intentions and behaviors among adolescent athletes. This finding is particularly pertinent to our study, as it suggests that these stereotypes might be a key factor contributing to the lower rates of participation observed among girls in football. The interplay of societal norms and gender stereotypes presents a complex barrier to female involvement in traditionally male-dominated sports, indicating the need for a nuanced understanding and approach to address these issues.

### 6.4 Age differences in football participation

Although the secondary school group reported higher scores than the primary school group on Attitudes, Past Behavior, and Future Behavior, the path analysis using structural equation modeling found that only the primary school group could significantly predict football participation. The differences in football participation between middle school and primary school students can be attributed to varying levels of academic pressure and availability of physical activity opportunities. Adolescents aged 13 to 16, typically in middle school, face greater academic pressure, with social factors like schools and parents focusing more on academic learning. This emphasis often results in reduced time and fewer opportunities for physical activities, adversely affecting their involvement in sports like football (Robertson-Wilson et al., 2007; Wang et al., 2017). In contrast, primary school students experience comparatively less academic pressure and have more opportunities for physical activities, including football. The reduced academic burden at this younger age allows for greater participation in sports, leading to a more significant influence of past behavior on their current involvement in football (Liu et al., 2016; Lu et al., 2017). Therefore, the contrasting academic environments and physical activity opportunities between these age groups explain the observed disparities in football participation.

### 6.5 Limitations and shortcomings

Although this study provides some insights into the factors influencing adolescent participation in football and gender differences based on the Theory of Planned Behavior, there are still some limitations and shortcomings.

Firstly, the sample, restricted to schools participating in a specific football program, may not adequately reflect the diverse landscape of campus football. Such a narrow focus could introduce selection bias and fail to represent the range of experiences influenced by varying resources, football culture intensity, and geographic or socioeconomic factors. The homogeneity in practices across these selected schools further hinders the generalizability of the findings. Therefore, interpretations of the results should be made with caution, and future studies should aim to include a more varied and representative sample to enhance accuracy and relevance.

Secondly, the study’s cross-sectional design limits its ability to establish causal relationships between variables. A longitudinal approach in future research could provide deeper insights into how these relationships evolve over time.

Thirdly, the current research is confined to primary and middle school students. Expanding future studies to encompass high school students could offer a more comprehensive view of how adolescent engagement in football changes with age.

Lastly, this study focuses primarily on internal factors affecting youth involvement in football. Future research should consider external factors, such as parental and peer support, to gain a holistic understanding of what drives and shapes adolescent participation in football sports. Such an approach would provide a more complete picture of the factors influencing youth in this field.

## 7 Conclusion

In summary, this study enhances the understanding of the Theory of Planned Behavior model by applying it to identify factors influencing adolescent participation in football sports. The findings underscore the significance of attitude, subjective norms, perceived behavioral control, and behavioral intention in this context. Additionally, the research delves into gender and age differences within the main pathways of the Theory of Planned Behavior model. It reveals that gender and age moderate the relationship between past behavior and behavioral intention, indicating notable differences in how boys and girls perceive and engage in football sports, with boys generally participating more actively than girls. This insight suggests the need for strategies to convert girls’ recognition of the value of football sports into more active participation. The outcomes of this study offer valuable insights for future research and interventions aimed at promoting adolescent engagement in football sports.

## Data availability statement

The raw data supporting the conclusions of this article will be made available by the authors, without undue reservation.

## Ethics statement

The studies involving humans were approved by Human Experimental Ethics Inspection of Guangzhou Sport University. The studies were conducted in accordance with the local legislation and institutional requirements. Written informed consent for participation in this study was provided by the participants' legal guardians/next of kin.

## Author contributions

XZ: Formal analysis, Investigation, Visualization, Writing – original draft. WH: Conceptualization, Methodology, Project administration, Resources, Supervision, Writing – original draft, Writing – review & editing.
